# Evaluation of the Role of the Activating Application Method in the Cold Sintering Process of ZnO Ceramics Using Ammonium Chloride

**DOI:** 10.3390/ma16010408

**Published:** 2023-01-01

**Authors:** Andrey V. Smirnov, Maxim V. Kornyushin, Anastasia A. Kholodkova, Sergey A. Melnikov, Artem D. Stepanov, Elena V. Fesik, Vilen V. Mnatsakanyan, Anton Smirnov, Yurii D. Ivakin

**Affiliations:** 1 Mobile Solutions Engineering Center, MIREA-Russian Technological University, 119454 Moscow, Russia; 2 Materials Science Department, Moscow Polytechnic University, 107023 Moscow, Russia; 3 Chemistry Department, M. V. Lomonosov Moscow State University, 119991 Moscow, Russia; 4 M.V. Lomonosov Institute of Fine Chemical Technologies, MIREA-Russian Technological University, 119454 Moscow, Russia; 5 Department of Education Informatization, Institute of Digital Education, Moscow City University, 129226 Moscow, Russia; 6 Laboratory of 3D Structural and Functional Engineering, Moscow State University of Technology “STANKIN”, 127055 Moscow, Russia

**Keywords:** oxide ceramics, zinc oxide, cold sintering, thermo-vapor treatment, microstructure

## Abstract

The influence of the method of applying the activating additive ammonium chloride and its concentration on the density and microstructure of zinc oxide ceramic obtained by cold sintering at 244 °C was investigated. The activating agent was applied by two methods: impregnation and subsequent autoclave treatment. When the powder was activated by the impregnation method, the crystal sizes remained at the initial level of 0.17–0.19 μm. After the autoclave treatment, the crystal sizes increased to 0.31–0.53 μm. Samples of cold sintering ZnO with relative density up to 0.96 and average grain sizes 0.29–0.86 μm were obtained. ZnO powders and ceramic samples were analyzed using SEM, TGA/DSC, and XRD to reveal the effect of the powder activation method and cold sintering conditions on the material microstructure. The effect of ammonium chloride concentration on grain growth and microstructure of ceramic samples is shown. It was found that the average grain size of ceramic samples with an increase in additive concentration passes through a minimum. In cold sintering of the autoclave activated powder, the effect of reducing the average grain size was observed. The results of this work are discussed on the basis of the idea of the solid-phase mobility of the crystal structure arising when interacting with an aqueous medium.

## 1. Introduction

Cold sintering (CS) is a well-known and an actively discussed low-temperature process for the consolidation of ceramics [1,2] and ceramic composites [3,4,5]. Despite its relative novelty and predominantly laboratory level of development [6], CS attracts significant research attention due to the great environmental and economic benefits of adoption in industry [7,8]. Recently, significant progress has been made in understanding the mechanisms of the process [9], some approaches to the implementation of CS have been scientifically substantiated [10], and basic intervals for varying process modes have been established for the most studied materials, such as ZnO, BaTiO_3_, and CeO_2_ [11].

However, despite the advances made, there are still many unknown aspects and unresolved research challenges in CS. Among the main ones, there is the choice of the type and concentration of the activating additive. A common approach is to choose them on the basis of solubility of the ceramic material in liquid media. Distilled or deionized water in an amount of 4–25 wt% is used if the material is soluble in water, such as NaCl [12], NaNO_2_ [13], Li_2_MoO_4_, and K_2_Mo_2_O_7_ [3]. If the sintered material is insoluble in water, aqueous solutions of acids or alkalis are usually used, for example, Ba(OH)_2_ for BaTiO_3_ [14]; 5–35 wt% NaOH 1–5 M for SiO_2_ [15], Zeolite [16] and Na_3.4_Sc_0.4_Zr_1.6_Si_2_PO_12_ [17]; and 1.6–60 wt% AcOH 1–17.5 M for ZnO [18,19,20], TiO_2_ [21], SnO [22], and Na_3.4_Sc_0.4_Zr_1.6_Si_2_PO_12_ [17]. Even for the most studied ZnO material, there is no clear understanding of the principle of selecting the type and concentration of an activating additive [11,23,24]. In addition, the issues of controlling the change in the quantity and state of the liquid/gaseous medium in the CS process remain unresolved [19,25]. In addition to the above, it remains unclear how liquid activating additives can be used. Little research has been published on this topic, and they were carried out with ZnO and mostly one additive: zinc acetate (Zn (CH_3_COO)_2_·2H_2_O) [26,27].

The decrease in temperature under the conditions of CS of oxides in most works [1,2] is explained by the role of liquid as a transport phase in the so-called “dissolution–precipitation” mechanism. In pressure dissolution theory, CS is believed to involve sequential dissolution steps at stressed grain contact points, then diffusion transfers along grain boundaries to open pore surfaces, and then deposition on the grains surface in the pore region. It is important that all this occurs under the action of chemical potential gradients aimed at minimizing excess surface energy of particles during compaction [28]. In the number of works [26,27,29,30], an alternative cold sintering mechanism has been proposed, according to which mass transfer and powder compaction occur due to the appearance of super pre-phase mobility of the crystal structure of oxides when interacting with an aqueous medium. The main ideas about solid-phase mobility developed during studying the influence of activating additives on mass transfer and the formation of fine crystalline powders during autoclave treatment of hydroxides or amorphous oxides in an aqueous medium at temperatures of 100–400 °C [31,32]. In the CS process, the powder also interacts with the aqueous medium, but to the fullest extent, the powder is pressed/compressed by mechanical force. The proximity of the conditions for interaction of the oxide with the aqueous medium during cold sintering and autoclave processing made it possible to consider the processes taking place from the standpoint of ideas about the low-temperature solid-phase mobility of the crystalline structure of oxides [29,30]. In the autoclave treatment of powders, two approaches are used: the first is the heating of the oxide powder in the activator solution; the second, one activator is preliminarily applied on powder by the impregnation method, and powder is worked on in the medium of water vapor or fluid [33]. These approaches were used in cold sintering of ZnO in a spark plasma sintering unit (SPS) with injection into a mold with a powder of 1.6 wt% of an aqueous solution of an activator or deionized water into a powder with a pre-applied activator [26]. It has been found that by rapidly heating the mold of the SPS unit, pre-application of the zinc acetate activating agent by impregnation provides sintered samples at 250 °C with a higher relative density than other methods of introducing the activator into the powder.

The present work continues the CS process study using the pre-addition of an ammonium chloride (NH4Cl) activator additive to the ZnO powder. Since there are currently no data on the effect of the NH_4_Cl additive method, two methods for bringing the activating additive have been selected in the present work: impregnation and thermal-vapor treatment, which result in different states of the ZnO powder [26]. The first studies showed a significant influence of these methods on the CS process using zinc acetate as an example. When the additive is applied by the impregnation method, its activating effect at CS can be explained within both mentioned mechanisms (i.e., “dissolution–precipitation” and solid-phase mobility). TVT leads to a change in the mass between crystals, a change in the dispersion of the powder, and the decomposition of the activating additive [31]. In this case, it is difficult to explain the dissolution–precipitation mechanism of compaction in CS of ZnO powder with zinc acetate subjected to TVT [26,27]. It is assumed that after decomposition of the additive during TVT, activation of the mobile state of the crystal structure is maintained. Due to this, the changed state of the crystals (solid-phase mobility) ensures CS of the powder without the additional use of an activator.

Based on studies of recrystallization of ZnO under TVT (100 °C < T < 450 °C), it was found that the addition of 0.3% to 3% by weight of NH_4_Cl leads to a pronounced growth of ZnO crystals and a change in their shape [31]. In the previous CS work, it has been shown that activation of the process by adding NH_4_Cl results in the formation of ceramics with a relative density greater than 0.9 and an average grain size of about half that of using acetate media in identical CS modes [29,30].

The purpose of the present work is to compare the CS activation of ZnO powder in two methods of pre-application of the NH4Cl additive: impregnation and TVT. The study is based on the idea of the influence of the components of the aqueous salt solution on the processes of mass transfer/redistribution between crystals of dispersed powder in the medium of water vapor or low-density aqueous fluid in the region of near and supercritical temperatures. The CS study is based on the idea that the formation of dense ceramics under these conditions is due to the influence of mechanical pressure on the mass transfer processes in the medium of water vapor or low-density fluid.

## 2. Materials and Methods

ZnO powder (JSC «Krasny Khimik», St. Petersburg, Russia) with average (median) particle size of 0.174 μm (mean 0.193 ± 0.002) was used in the work. The sintering activating agent was NH_4_Cl ammonium chloride (AmCl). All reagents had a purity of >99% by weight. The reference designations of the samples are given in Table 1.

To apply the activating agent by impregnation, 20 g of ZnO powder was mixed with 30 mL of an aqueous activator solution when treated in an ultrasonic bath. The resulting mass, after drying for 12 h at 70 °C, was triturated in an agate mortar and sieved through a 300 μm capron sieve. The composition of the samples after application of AmCl by the impregnation method is shown in Table 2.

The second option for ZnO activation was TVT treatment of ZnO powder with AmCl deposited in a vapor medium at 220 °C in a laboratory autoclave. The 1–5 g powder was poured into a Teflon (PTFE) container, which was placed in a 17 mL autoclave on a stand. Outside the powder container, distilled water was poured onto the bottom of the autoclave in an amount of 20% of the free volume of the autoclave. The autoclave was sealed, heated, and held at 220 °C for 20 h. At the same time, heating and isothermal exposure of the powder took place in a vapor medium. The features of TVT have been described in detail in [27,29,30].

**Figure 1:**
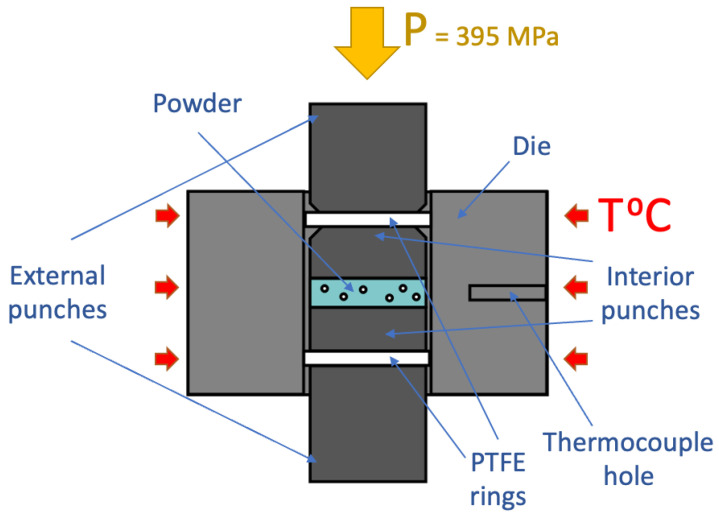
CS setup scheme.

CS was performed in a steel mold with induction heating (Figure 1). The mold contained four punches (11 mm in diameter), between which there was a ZnO powder in the middle, and between the other punches, there were PTFE O-rings. O-rings were used to prevent water from extruding and evaporating through gaps in the mold during pre-pressing and during CS mixing.

ZnO powder in an amount of 1 g was poured into the mold, and 0.2 mL of distilled water was added with stirring. Then, the second pair of punches with or without a sealing ring were added (Figure 1). The thermocouple was placed in the cavity of the mold adjacent to the powder. The molding was conducted on a P–50 hydraulic press providing a pressing force of up to fifty tons. A mold with a heater was installed along the axis of the hydraulic press (Figure 1). The shrinkage of the powder in the mold was controlled by measuring the axial displacement of the lower platform of the hydraulic press using a mechanical clock-type movement indicator (with a division price of 10 μm) mounted on the frame. CS mode was selected based on the results of [29,30]: sintering temperature 244 °C, heating time to sintering temperature of 40 min, holding time of 60 min, and PTFE O-rings. The heating was started when the pressing was 395 MPa. The used pressing force, at which no deformation of the mold tooling occurred, was selected in the preliminary tests.

Powder morphology and microstructure of ceramics were examined using an electron microscope JSM–6390 LA (JEOL Ltd., Tokyo, Japan). The crystal size distribution of the powder and grains of the CS ceramic samples was determined by analyzing scanning electron microscope (SEM) images. Measurements were made using Image-Pro Plus software (version 4.5, Media Cybernetics, Inc., Rockville, MD, USA). On the SEM image, the particle size was measured, the contour of which is reliably determined. Measurements covered particles of the upper layer and partially of the lower layer of powder. On the fractured surface of the ceramic samples, grains of the upper layer were measured. In the case of isometric grains, the diameter was measured. For non-dimensional grains, the area of the grain was measured, which was converted into an equivalent diameter [34]. As a characteristic of the crystal size, the average/mean size (d_mn_) and the median measured values (d_md_) were used, which gave a more accurate result for an asymmetric distribution, since it was not affected by emissions in the set of measurements in the case of small volumes of statistical sample.

Thermal analysis (TGA/DSC) of powder and ceramic samples was carried out in STA 449 C Jupiter thermal analyzer (Erich NETZSCH GmbH & Co. Holding KG, Selb, Germany). The samples were heated in argon with the rate of 10 °C/min from 40 to 800 °C. X-ray diffraction analysis of the initial and activated powders as well as of ceramic samples was conducted by means of X-ray diffractometer XRD 6000 (Shimadzu Corp., Kyoto, Japan). A high-resolution Image Plate Huber G670 camera was used, CuKα1 radiation, λ = 1.540598 Å, Ge (111) monochromator, angular range 3000–100,300° 2θ°, and pitch 0.005° 2θ.

Relative density of ceramics was determined at 20 ± 2 °C and 60 ± 5% relative humidity by the Archimedes method. Kerosene was used as a saturating medium, since sample destruction could occur in distilled water. Partial fracture at the edges of the sample when determining density in a liquid medium increased the error of the measurement result.

## 3. Results

**Figure 2:**
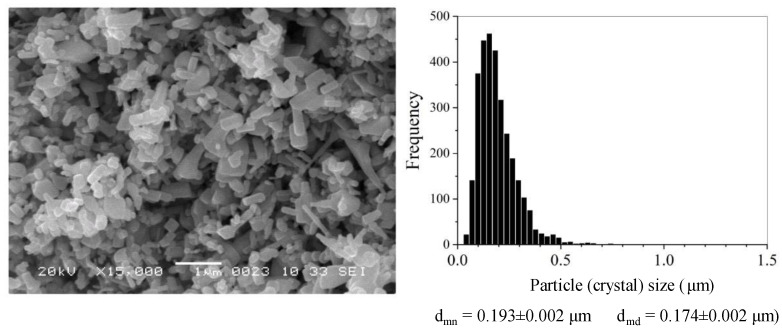
SEM image and crystal size distribution of ZnO powder at baseline.

**Figure 3:**
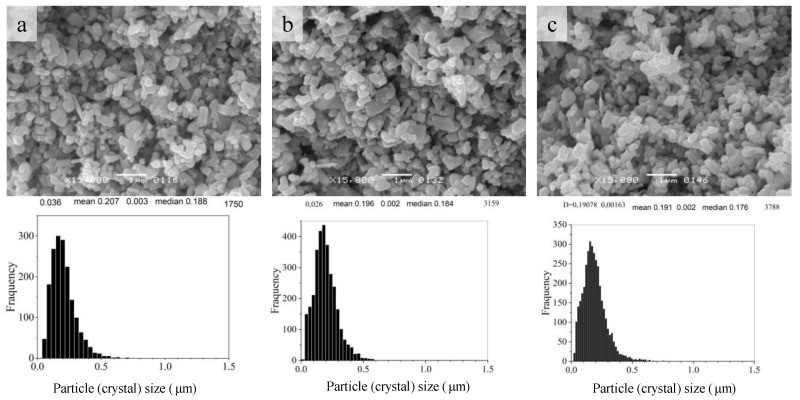
SEM image and crystal size distribution of ZnO powder after activation by the impregnation method with additive AmCl: 0.3% (**a**), 1% (**b**), and 3% (**c**).

The ZnO stock powder has crystals of various habituses with dimensions mainly less than 0.5 μm (Figure 2). After the AmCl additive is applied by the impregnation method, thin elongated crystals disappear, but the size range of the powder crystals does not change. However, when comparing histograms of crystal size distributions (Figure 3), with an increase in the content of the additive in the dispersed composition of the powder, fine crystals appear. These crystal particles on the histogram of the size distribution (Figure 3) correspond to the appearance and growth of the shoulder on the left wing of the histogram.

**Figure 4:**
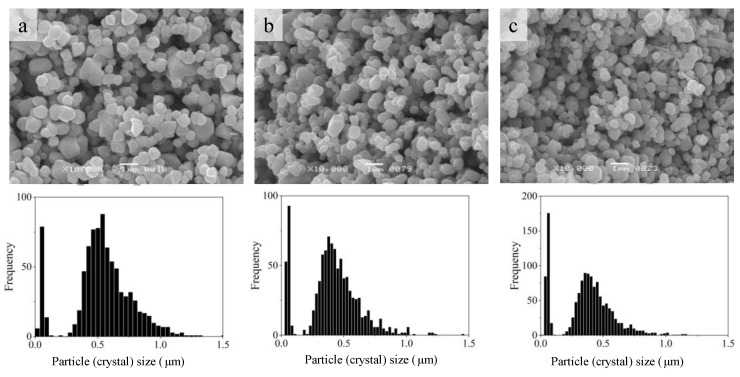
SEM image and crystal size distribution of ZnO powder activated by the TVT method at content of additive AmCl: 0.3% (**a**), 1% (**b**), and 3% (**c**).

TVT of the powder with the applied additive AmCl leads (Figure 4) to the growth of crystals of the powder and the isolation of fine crystals into a separate component (a fine component of the dispersed composition of the powder) concentrated in a narrow range of 0–0.2 microns on the axis of crystal size. Moreover, with an increase in the content of AmCl additive, the relative proportion of crystals of the fine component increases.

**Figure 5:**
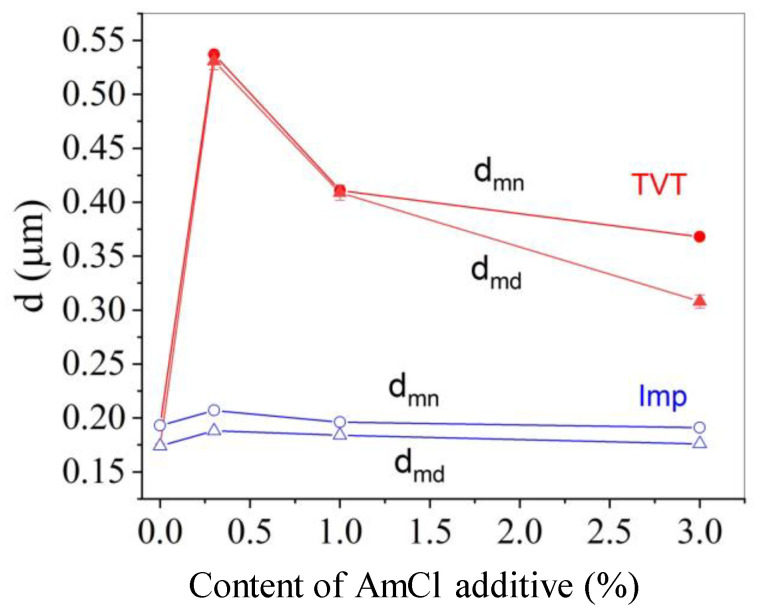
Dependence of the average particle (crystal) size of ZnO powder on the concentration of AmCl and the activation method: impregnation (Imp, blue line) and TVT (red line). Letters indicated: d_mn_—average size (circles); d_md_—median value (triangles).

Figure 5 shows the change in mean and median with the addition of two powder activation methods. As the number of additive increases, the average crystal size decreases with both activation methods. In the case of impregnation, this is due to an increase in the proportion of the fine component. Activation of the powder by the TVT method results in crystal sizes twice as large. In this case, the overall average crystal size with an increase in the activator content is more influenced by a decrease in the crystal size of the main component. It can be noted that the average crystal sizes in mean and median formats vary equally.

**Figure 6:**
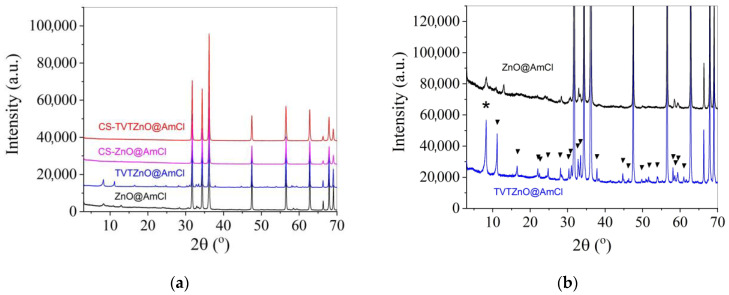
XRD patterns of powders (**a**) activated with 3% AmCl additive and obtained samples of CS ceramics; (**b**) the triangular icon marks reflexes corresponding to Zn_5_(OH)_8_Cl_2_*H_2_O (JCPDS 7–155), and an unidentified phase is marked with an asterisk.

Figure 6a shows XRD patterns of powders activated with 3% AmCl and their CS ceramic samples. Against the background of intensive ZnO reflexes on diffraction patterns of activated powders (ZnO@AmCl and ZnO@AmClTVT), there are small reflexes of impurity phases. These impurity phases disappear after CS (CS–ZnO@AmCl and CS–ZnO@AmClTVT). In Figure 6b, XRD patterns of samples with impurity phases are shown on a larger scale. The triangular icon marks reflexes corresponding to zinc hydroxide monohydrate Zn_5_(OH)_8_Cl_2_·H_2_O (JCPDS 7–155). An unidentified phase is marked with an asterisk. From the comparison of the patterns, it follows that after impregnation of the powder with AmCl solution, traces of impurity phases appear and after TVT their presence increases markedly. However, under CS conditions, the impurity phases disappear.

**Figure 7:**
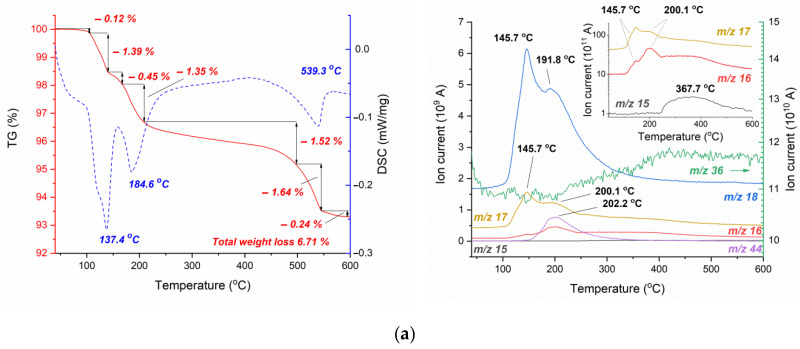

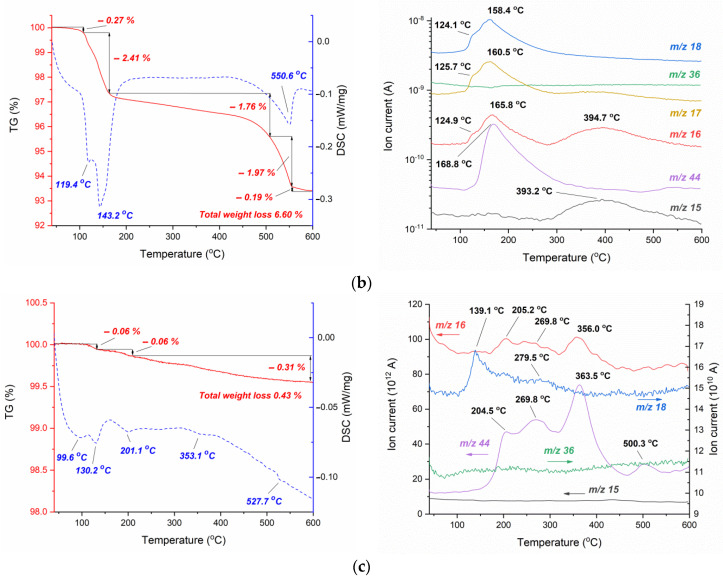
TGA/DSC (left) and MS (right) curves for ZnO@AmCl(3%): (**a**) TVTZnO@AmCl(3%); (**b**) CSTVTZnO@AmCl(3%); and (**c**) samples. In TG/DSC graphs: red—TG curves; blue lines—DSC curves. In MS graphs, colored lines corresponded to the indicated *m*/*z* values.

Figure 7 shows the results of thermogravimetric analysis of ZnO@AmCl powder (with 3% AmCl) and TVTZnO@AmCl powder (with 3% AmCl), as well as the resulting ceramic sample CSTVTZnO@AmCl. Weight loss of 0.12% at 105 °C is associated with desorption of weakly bound water. The weight loss of the ZnO@AmCl sample in the range from 105 °C to 208 °C (Figure 7a—powder activated by the impregnation method) has at least three stages (1.39%, 0.45%, and 1.35%—only 3.19%) with a two-stage water release and an endoeffect at 137 °C. The second endoeffect (at 185 °C) in this temperature region with the release of water and CO_2_ can be associated with the decomposition of surface hydroxocarbonates. In addition, ammonia is released, which is evidenced by the maxima MS 17 and 16 *m*/*z* in the absence of a signal from 15 *m*/*z* (Figure 7a, insert). Ammonia is recovered by reacting the adsorbed additive NH_4_Cl and ZnO to form ZnCl_2_. Then, the slow decline of MS curves for masses 17 and 16 *m*/*z* in the temperature region of 250–500 °C is accompanied by a small contribution of the exoeffect, noticeable in the growth of the DSC curve in the temperature region of 300–500 °C (Figure 7a). At a higher temperature, the resulting ZnCl_2_ sublimates with a weak release of HCl (36 m/e in Figure 7a) and an endoeffect with a maximum at 539 °C. ZnCl_2_ sublimation is not recorded in mass spectrometric gas flow analysis due to condensation on cold walls. TVT changes the decomposition of surface compounds during thermal analysis of the TVTZnO@AmCl sample (Figure 7b). A slight decline in the MS curve of 36 *m*/*z* indicates the isolation at T < 150 °C of a small amount of HCl weakly bound on the surface of ZnO crystals. As can be seen from the TGA/DSC data, the release of H_2_O and CO_2_ has two stages with a mass loss of 2.41%. However, the main process proceeds in a narrower temperature range from 109 °C to 164 °C and slowly decays to 500 °C. The change in the isolation of water and CO_2_ during heating of the TVTZnO@AmCl sample is due to the structuring of the Zn_5_(OH)_8_Cl_2_·H_2_O phase, the formation of which is observed during impregnation. The substantially equal residual weight of 93.30% and 93.42% for the impregnated and TVT samples, respectively, indicates the decomposition of the surface compounds that preceded the formation of the Zn_5_(OH)_8_Cl_2_·H_2_O phase and the resulting phase. The endoeffect at 119 °C is associated with the first stage of water release and the formation of Zn_5_(OH)_8_Cl_2_. The co-release of water and CO_2_ with the maximum endoeffect at 143 °C is probably caused by the decomposition of Zn_5_(OH)_8_Cl_2_. It is important to note here that the second endoeffect observed in the previous case at 185 °C has disappeared. It was associated with the reaction between NH_4_Cl and ZnO in impregnated samples. With TVT activation, instead of NH_4_Cl, Zn_5_(OH)_8_Cl_2_·H_2_O was formed in the powder. A wide synchronous maximum of MS curves 16 *m*/*z* and 15 *m*/*z* with a slight change of 17 *m*/*z* of about 400 °C indicates methane release. Weight loss of 1.97% at temperatures above 500 °C with an endoeffect at 550 °C is similar to that observed for the impregnated sample. It is also associated with ZnCl_2_ sublimation.

A small total weight loss of 0.45% of the ceramic sample CSTVTZnO@AmCl (Figure 7c) occurs when adsorbed water and SO_2_ are isolated. The absence of appreciable mass loss effects corresponds to the XRD degradation of impurity phases during CS.

**Figure 8:**
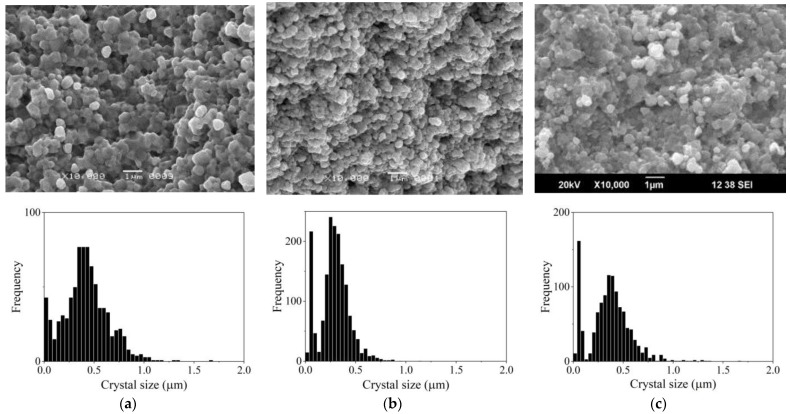
Microstructure and grain size distribution in CS samples of powder activated by impregnation with additive 0.3% (**a**), 1% (**b**), and 3% (**c**) AmCl.

**Figure 9:**
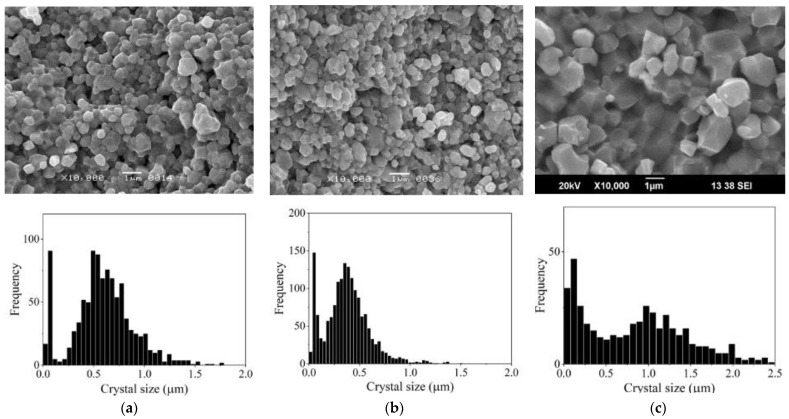
Microstructure and grain size distribution in CS samples of powder activated by TVT with additive 0.3% (**a**), 1% (**b**), and 3% (**c**) AmCl.

Histograms of crystal grain size distribution in ceramic samples are shown in Figure 8 for CS ZnO powder activated by impregnation and Figure 9 for powder with TVT activation.

When comparing the histograms in Figure 3 and Figure 8, it can be seen that under CS conditions, the size of the main component of crystalline grains increased, and a fine component isolated in a narrow size range appeared. The result of crystal mass redistribution is similar to the change in powder dispersion during TVT (Figure 4) with CS at 244 °C lasting 40 min and TVT at 220 °C lasting 20 h. A fine powder component with TVT activation (TVTZnO@AmCl) and its size range are preserved in ceramic CS samples with 0.3% and 1% addition (Figure 4a,b and Figure 9a,b) with a slight change in the size of the grains of the main component. In the case of an addition of 3% after CS, the size of crystalline grains, both the main component and fine component (Figure 4c and Figure 9c), sharply increased.

**Figure 10:**
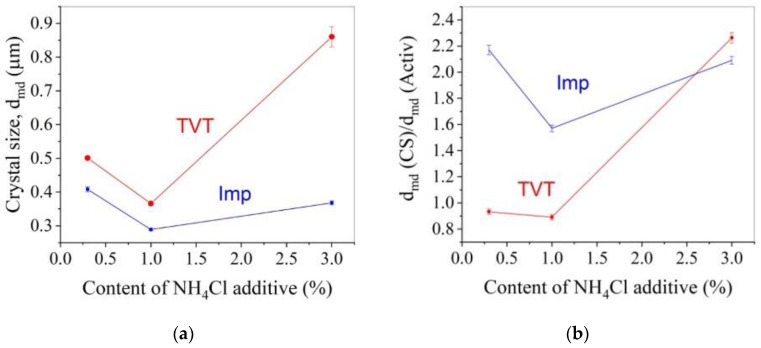
Dependence of average grain size (**a**) and relative change in average crystal size (**b**) of CS samples on concentration of AmCl additive and method of ZnO powder activation: red line, circles-TVT method (indicated as TVT); blue line, squares-impregnation method (indicated as Imp).

Figure 10a shows the change in average crystal size during CS with increasing additive content. The average size of crystalline ceramic grains varies from 0.29 to 0.86 μm, in which the minimum grain was obtained by activating the powder by the impregnation method, and the largest grain and most of the size range from 0.366 to 0.86 μm belong to the TVT activation method. Figure 10b shows the change in the average size of crystalline grains of ceramic samples relative to the average size of the crystals of the used powder (according to the ordinate, the ratio of the average sizes of ceramic grains and powder crystals is shown). When activated by impregnation, the size of crystalline grains increases 1.5–2 times during CS with a weak dependence on the content of the additive. Crystal growth under CS conditions is due to the same effect of the medium on mass transfer processes at CS and TVT and corresponds to the ratio of average crystal particle sizes in Figure 5.

During CS, there is a relative decrease in the average crystal size of the TVT-activated powder (Figure 10b) in the low additive content area. This change also corresponds to the course of the relationship in Figure 5, but the shift of the left wing of the main component in the histogram (Figure 4 and Figure 9) noted above is more pronounced. In contrast to this relationship, increasing the additive content to 3% results in a sharp increase in crystal size at CS (Figure 10b). This effect should be associated with the action of compressing the powder by mechanical force.

**Figure 11:**
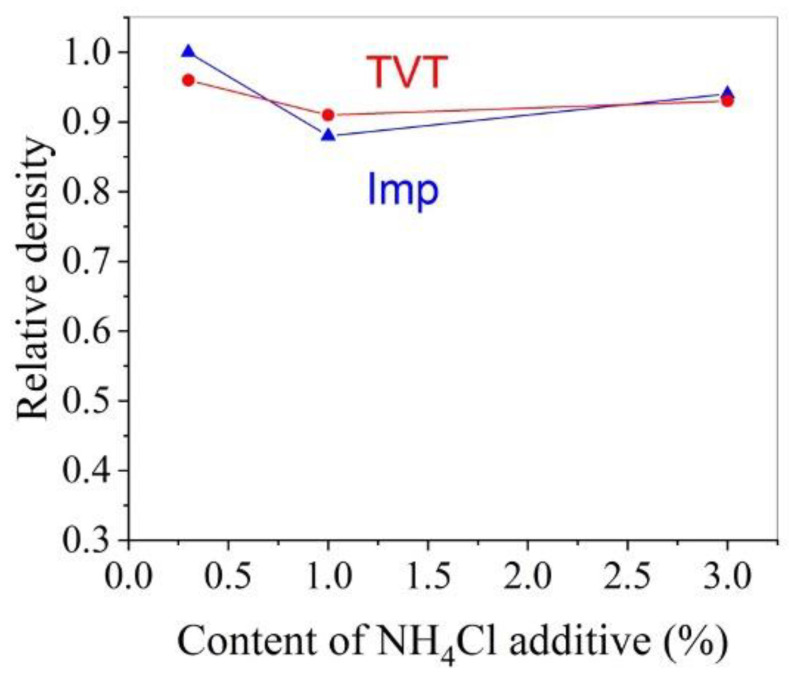
Effect of AmCl content and powder activation method on the relative density of CS ceramic samples: red line, circles-TVT method (indicated as TVT); blue line, triangles - impregnation method (indicated as Imp).

Figure 11 shows the dependence of the relative density of CS ceramic samples on the content of ammonium chloride additive in two powder activation methods. It can be seen that both methods of activating the powder make it possible to obtain high-density ceramics over the entire range of AmCl concentrations used.

## 4. Discussion

Previously, the experiments of ZnO cold sintering in pure water performed poor effectiveness because of low interaction rate of ZnO and H_2_O [26]. Application of AmCl additive by the impregnation method and then treating the powder in the TVT conditions activate cold sintering of ZnO powder to form high-density ceramics. When the ZnO powder is impregnated with AmCl solution, the formation of impurity phases occurs in insignificant amounts (at the trace level), and in the water vapor medium at 220 °C during TVT, the formation of impurity phases is recorded by the XRD method (Figure 6b). Zinc hydroxide monohydrate Zn_5_(OH)_8_Cl_2_·H_2_O was found in their composition. The formation of this phase under similar conditions has been reported in [25,30,35]. This is a layered compound with a high interlayer distance (0.79 nm), which could be occupied by H_2_O or CO_2_ molecules [36]. Simultaneous ejection of H_2_O or CO_2_ observed in TGA of TVTZnO@AmCl is associated with the decomposition of Zn_5_(OH)_8_Cl_2_·H_2_O.

The thermal decomposition of Zn_5_(OH)_8_Cl_2_·H_2_O is affected by the humidity of the surrounding atmosphere [36]. In this work, the decomposition commenced above 100 °C with elimination of water when Zn_5_(OH)_8_Cl_2_·H_2_O dehydrated to Zn_5_(OH)_8_Cl_2_. Then, in a range of 161–197 °C, Zn_5_(OH)_8_Cl_2_ transformed to amorphous ZnO·ZnCl_2_·2H_2_O. Decomposition of ZnO·ZnCl_2_·2H_2_O is affected by the humidity. In a humid medium, it transforms into ZnO with elimination of HCl, while in a dry atmosphere, above 225 °C, water and ZnO·ZnCl_2_ are formed. Above 400 °C, ZnCl_2_ was reported to volatilize.

From the Figure 7a,b, ZnCl_2_ was volatilized on heating ZnO@AmCl and TVTZnO@AmCl samples in argon above 450 °C. In the case of ZnO@AmCl, the formation of ZnCl_2_ accompanied by ammonia elimination occurred at about 200 °C when NH_4_Cl interacted with ZnO. This result corresponded to that reported in [37]. A weak exothermal effect at about 400–450 °C (Figure 7a) could be attributed to the crystallization of ZnCl_2_, which evaporated above 450 °C. ZnCl_2_ evaporation on the heating of TVTZnO@AmCl was caused by Zn_5_(OH)_8_Cl_2_ decomposition and the formation of intermediate ZnO·ZnCl_2_. Due to this, the temperature of ZnCl_2_ evaporation appeared to be higher (Figure 7a,b). A higher amount of ZnCl_2_ (1.97% and 1.64%) is explained by the partial decomposition of NH_4_Cl on heating the ZnO@AmCl sample. Methane detection during TGA of TVTZnO@AmCl pointed to catalytic activity on the ZnO·ZnCl_2_ surface in relation to CO_2_ and H_2_O. According to our data, Zn_5_(OH)_8_Cl_2_·H_2_O remains stable at a temperature of TVT 220 °C and decomposes under CS conditions at 244 °C. The result is a ceramic free of volatile impurities (Figure 7b).

Comparison of crystal size distributions (Figure 3, Figure 4, Figure 8 and Figure 9) leads to the conclusion that the mass redistribution between the crystals due to the influence of the wet medium begins already at 70 °C during the drying process of the powder impregnated with the AmCl solution. The first process is the formation of new crystals of the fine component (Figure 3). With TVT powder with an AmCl additive, the formation of crystals of the fine component is completed, and diffusion redistribution processes occur with the growth of crystals of the original powder. Crystals of the fine component are not involved in these processes. The causes and mechanism of small crystal formation are not clear and require further investigation. These special properties also appear in the formation of ceramics—crystals of a fine component remain at the boundaries between the growing crystalline grains of ceramics.

The growth of crystals of the main component of ZnO powder occurs by two mass transfer mechanisms at temperatures above 70 °C. As previously described [29,30,31] by one mechanism, slow crystal growth (Figure 10) occurs with diffusion spreading of the mass of crystals with increased solid-phase mobility. Increased solid-phase mobility of the crystal structure appears under the influence of an additive that activates the exchange interaction of crystals with an aqueous medium. The second mechanism leads to rapid crystal growth. It is associated with the coalescence of neighboring crystals due to the disappearance of the border with sufficient crystallographic correspondence [38]. The coalescence mechanism begins to appear when a certain threshold for the content of the activating additive is exceeded from 1 to 3%. This means that after exceeding a certain threshold of the content of the activating additive, it becomes possible to achieve a crystallographic correspondence. When the content of the activating additive is low, the pressing force brings together and deforms the fine crystals with the movable structure, resulting in the formation of a dense ceramic (Figure 8c and Figure 9a,b). The threshold content of the additive is associated with the need to reorient neighboring crystals to a crystallographic correspondence, which, with their dense packaging, cannot occur due to the rotation of the crystals. The increase in the content of the activating additive causes an increase in the structural mobility of the crystals and their diffusion rearrangement. At the same time, the degree of influence of deformation caused by mechanical force is reduced.

As a result of coalescence of a group of neighboring crystals, crystalline grains are formed with forced cutting (Figure 9c) and filling the intergranular space [30]. Crystals of the fine component do not participate in coalescence. Their structure does not have solid-phase mobility. For an unknown reason, the AmCl additive does not activate their interaction with the aqueous medium. Crystal size of fine component depended on structural mobility of the main component. When the size of the main component sharply increased with the increase in the additive amount, the size of the fine component grew as well (Figure 9). This effect was revealed for ZnO recrystallization in TVT conditions [31] and was observed during CS [30]. Crystals of the main component of ZnO@AmClTVT powder, during TVT, grow and acquire a more perfect structure due to the ordering process when interacting with the medium [30]. The traces of activator remained in their structure, which was evidenced by the formation and sublimation of ZnCl2 at 450–550 °C during the thermal analysis of the activated powders. The trace amounts of the activator provided sufficient mobility in water medium during CS so that mass transfer processes are restored under cold sintering conditions, even though pure water without an activator was added to the powder. This is like the long-term preservation of mass transfer processes when storing ZnO powder (synthesized in an aqueous acetate medium) in a humid atmosphere and at room temperature [39].

The difference between powder activation by impregnation and TVT is that during TVT activation, the main slow process of mass transfer with crystal growth has already passed, and under CS conditions, the coalescence of crystals under the action of mechanical pressure with an increased content of the activator prevails (Figure 10a). The result is ceramics with relatively large grains. When the powder is activated by the impregnation method, a slow mass transfer does not have time to lead to the growth of large crystals in a short time under CS conditions (Figure 10a). However, the crystals increase by about a factor of two (Figure 10b). Densification of ceramics at a low content of the activator is achieved, as noted above, due to the deformation of crystals with a mobile structure. As a result, small parts are separated from large crystals of the main component of the TVTZnO@AmCl powder, and the average size of crystalline grains decreases (histograms in Figure 4a,b and Figure 9a,b, as well as Figure 10b). Grain size reduction in CS ceramics was also found in [25]. The falling branches of dependencies in the range of low additive content in Figure 10a,b are due to an increase in the improvement of the crystal structure with an increase in the content of the additive. With an increase in the perfection of the structure, the intensity of the exchange of water molecules with the medium decreases, the solid-phase mobility, and mass transfer by the surface spreading mechanism (slow growth mechanism) decrease. In addition, large crystals of the main component of the TVTZnO@AmCl powder are more easily crushed, losing structural mobility. However, in the region of a higher content of the additive, the mobility of the structure covers the volume of crystals and the probability of their coalescence increases. Thanks to this, the dependence branches in Figure 10a,b rush up. The described processes also affect the relative density of ceramics, the dependence of which on the additive content also has branches of different directions.

## 5. Conclusions

Two methods of introducing an additive from 0.3 to 3 wt% ammonium chloride by impregnation and autoclave thermal-vapor treatment led to a different state, dispersion, and activity of ZnO powder during cold sintering. The average size of 0.174 microns of the initial crystalline powder particles after the ammonium chloride addition increased to 0.176–0.53 microns. Cold sintering of the activated powder at a temperature of 244 °C in the presence of distilled water made it possible to obtain ceramics with a relative density of up to 0.96. The grain size was in a range of 0.29–0.86 μm. When the powder is activated by impregnation or TVT, the grains of ceramics vary their sizes between 0.29 and 0.41 μm or 0.366 and 0.86 μm. The discussion of the processes occurring during thermal-vapor treatment and cold sintering of ZnO powder is based on the idea of the appearance of solid-phase mobility of the crystal structure when interacting with an aqueous medium. It is concluded that the application of mechanical pressure to the powder leads to the formation of the ceramic’s dense microstructure and different grain sizes by two mechanisms: the first, due to deformation of crystals with mobile structure and moderate crystal growth; and the second, by crystals coalescence with the formation of large grains when structural mobility occurred in their bulk on the increase in the activator amount.

## Figures and Tables

**Table 1:** The sample’s reference designations.

	Before CS	After CS
Impregnation	ZnO@AmCl	CS-ZnO@AmCl
TVT	TVTZnO@AmCl	CS-TVTZnO@AmCl

**Table 2:** Content of AmCl additive in ZnO when applied by the impregnation method.

	Content 1	Content 2	Content 3
wt%	0.3	1	3
mol%	0.456	1.52	4.56

## Data Availability

The data presented in this study are available on request from the corresponding author after obtaining the permission of an authorized person.
